# Characteristics and prognosis of isolated aortic valve infective endocarditis in patients with bicuspid aortic valves: a propensity matched study

**DOI:** 10.3389/fcvm.2023.1304957

**Published:** 2023-12-14

**Authors:** Yohann Bohbot, Seyhan Denev, Rossella M. Benvenga, Mary Philip, Hector I. Michelena, Rodolfo Citro, Gilbert Habib, Christophe Tribouilloy

**Affiliations:** ^1^ Department of Cardiology, Amiens University Hospital, Amiens, France; ^2^ UR UPJV 7517, Jules Verne University of Picardie, Amiens, France; ^3^ Division of Cardiology, Cardiovascular and Thoracic Department, San Giovanni Di Dio E Ruggi d, Aragona University Hospital, Salerno, Italy; ^4^ Department of Cardiology, Hôpital de la Timone, Aix-Marseille University, Marseille University Hospital, Marseille, France; ^5^ Department of Cardiovascular Medicine, Mayo Clinic, Rochester, MN, United States

**Keywords:** bicuspid aortic valve, tricuspid aortic valve, infective endocarditis, survival, surgery

## Abstract

**Introduction:**

Bicuspid aortic valve (BAV) is the most common congenital heart disease with an increased risk of infective endocarditis (IE). Few data are available on isolated native BAV-IE. The aim of this study was to compare patients with tricuspid aortic valve (TAV) IE and BAV-IE in terms of characteristics, management and prognosis.

**Material and methods:**

We included 728 consecutive patients with IE on isolated native aortic valve in 3 centres: Amiens and Marseille Hospitals in France and Salerno Hospital in Italy. We studied in hospital and long-term mortality before and after matching for age, sex and comorbidity index. Median follow-up was 67.2 [IQR: 19–120] months.

**Results:**

Of the 728 patients, 123 (16.9%) had BAV. Compared with patients with TAV-IE, patients with BAV-IE were younger, had fewer co-morbidities and were more likely to be male. They presented more major neurological events and perivalvular complications (both *p* < 0.05). Early surgery (<30 days) was performed in 52% of BAV-IE cases vs. 42.8% for TAV-IE (*p* = 0.061). The 10-year survival rate was 74 ± 5% in BAV-IE patients compared with 66 ± 2% in TAV-IE patients (*p* = 0.047). After propensity score matching (for age, gender and comorbidities), there was no difference in mortality between the two groups, with an estimated 10-year survival of 73 ± 5% vs. 76 ± 4% respectively (*p* = 0.91).

**Conclusion:**

BAV is a frequent finding in patients with isolated aortic valve IE and is associated with more perivalvular complications and neurological events. The differences in survival with TAV-IE are probably related to the age and comorbidity differences between these two populations.

## Introduction

The characteristics and prognosis of infective endocarditis (IE) in patients with a bicuspid aortic valve (BAV) are poorly described (1) as initial reports included few patients and provided limited prognostic data (2). About a decade ago, our teams reported in a small pilot study that patients with BAV-IE are younger and have fewer comorbidities than those with tricuspid aortic valve IE (TAV-IE) (3). This study on native valve IE (including multivalvular IE) also highlighted that BAV-IE are frequently complicated by perivalvular complications, but no differences was observed with TAV-IE in terms of in-hospital mortality and 5-year age-adjusted survival (4). In this context, we aimed to update and complement this pilot study by including a larger number of patients from 3 tertiary centres, comparing BAV-IE and TAV-IE after matching, and focusing only on isolated aortic valve IE.

## Methods

We retrospectively enrolled patients with a diagnosis of isolated native aortic valve definite IE referred to two French (Amiens and Marseille) and one Italian tertiary centres (Salerno) between January 2000 and December 2019. Patients were subsequently classified into two groups according to aortic valve anatomy. Diagnoses of BAV were made on the short-axis view and were confirmed by another imaging technique when there was doubt about the diagnosis (4). The study was approved by an independent ethics committee and conducted in accordance with institutional policies, national legal requirements, and the tenets of the revised Declaration of Helsinki. Trial registration number: NCT03211975.

Transthoracic and transesophageal echocardiographic studies were performed on all patients during the acute phase of IE. Echocardiographic data included the confirmation of isolated aortic localization, presence, mobility, and maximal length of vegetations (vegetation length was measured in various planes and the maximal length was used), and the presence and localization of perivalvular complications, defined as an abscess, pseudoaneurysm, or fistula, according to accepted definitions (1). BAV disease was previously known or incidentally diagnosed during hospitalization. The diagnosis of embolism was based on either clinical signs or on data derived from noninvasive procedures (cerebral and thoracoabdominal computed tomography performed in the absence of severe renal insufficiency or hemodynamic instability). A neurological event was defined by the development of a confirmed ischemic stroke, hemorrhagic stroke, cerebral abscess, or a cerebral mycotic aneurysm. Information on follow-up was obtained yearly over the same period for the entire cohort. In-hospital mortality was defined as the occurrence of death during the initial hospitalization or within 1 month of diagnosis, and long-term mortality was defined as the occurrence of death during follow-up. Recurrence of IE was defined as the appearance of a new IE after the previous one had been considered cured following appropriate antibiotic treatment, at least three months after the first episode. Median follow-up was 67.2 [interquartile range (IQR): 19–120] months.

SPSS version 18.0 software (IBM, Armonk, New York) was used for all statistical analysis. Continuous variables are expressed as the mean value ± one standard deviation or medians (25th and 75th percentiles) and categorical variables as frequency percentages and counts. Factors associated with BAV IE and those associated with early surgery were identify using multivariable logistic regression (all variable with a *p* value < 0.10 were included in the analysis). Survival rates ± standard errors were estimated using the Kaplan–Meier method and compared by two-sided log-rank tests. The imbalance in baseline variables between BAV-IE and TAV-IE patients in terms of age, sex and Charlson index was reduced by use of propensity scores. We estimated propensity scores for each of the 728 patients using a multivariate logistic regression model, as previously described (5). Propensity scores were used to match each BAV-IE patient with a unique control with a propensity score within 2%. Then, 1:1 propensity score matching using a 5-to-2 digits greedy matching without replacement technique was used to create matched samples for analysis: each patient with BAV-IE was first matched with another patient with TAV with a similar 5-digit propensity score, and matched patients were removed from the database. This procedure was repeated in the remaining patients with successive matching by 4-, 3-, and 2-digit scores (6). One hundred and nine (89%) of the 123 patients with BAV-IE were successfully matched. After matching, the mean propensity scores between patients with BAV-IE (0.7517220 ± 0.1439) and those with TAV-IE (0.7519218 ± 0.1440) were not statistically different (*p* = 0.99). A standardized difference of <0.1 was used to assess covariates balance achieved between the matched samples. The limit of statistical significance was *p* < 0.05. All tests were two tailed.

## Results

### Entire cohort

We included 728 patients [123 with BAV (16.9%)]. Patients with BAV-IE were younger, more often males and had fewer comorbidities than those with TAV-IE. Neurological events were more frequent in BAV-IE but no differences were observed regarding embolic complications and congestive heart failure between groups. Vegetations were more frequently observed in TAV-IE, whereas perivalvular complications were more than twice as common in BAV-IE (Table 1). By multivariable logistic regression analysis, younger age [OR(95%CI) = 0.95(0.93–0.97)], male sex [OR(95%CI) = 2.38(1.26–4.48)], neurological events [OR(95%CI) = 1.85(1.06–3.23)], negative blood cultures [OR(95%CI) = 1.95(1.07–3.57)] and perivalvular complications [OR(95%CI) = 2.04(1.24–3.36)] were independently associated with BAV-IE (all *p* < 0.030) (Table 2).

**Table 1:** Comparison of demographic, clinical and biological characteristics between infective endocarditis on tricuspid aortic valves vs. bicuspid aortic valves.

Variables	TAV-IE (*n* = 605)	BAV-IE (*n* = 123)	*p*
General characteristics			
Age (years)	62 ± 14	48 ± 16	**<0**.**001**
Male sex (%. *n*)	76.2 (461)	87.8 (108)	**0**.**005**
Hypertension (%. *n*)	30.4 (184)	15.4 (19)	**0**.**001**
Diabetes (%. *n*)	20.5 (124)	7.3 (9)	**0**.**001**
Previous myocardial infarction (%. *n*)	7.8 (47)	3.3 (4)	0.082^a^
History of infective endocarditis (%. *n*)	3.8 (23)	4.9 (6)	0.578
Charlson comorbidity index (excluding age)	2.79 ± 2.1	1.58 ± 1.3	**<0**.**001**
Clinical features			
Major neurological event (%. *n*)	14 (85)	22 (27)	**0**.**027**
Embolic event (%. *n*)	44 (266)	45.5 (56)	0.751
NYHA class (%. *n*)			
I–II	76.2 (461)	72.4 (89)	0.366
III–IV	23.8 (144)	27.6 (34)	
Biology			
Plasma creatinine (µmol/L)	133.8 ± 137.9	121.3 ± 118	0.439
White blood cells (10^3^/mm^3^)	11.0 ± 11.5	10.5 ± 4.5	0.714
Microbiology			
Staphylococcus spp (%. *n*)	24 (145)	17.1 (21)	0.097
Streptococcus spp (%. *n*)	33.2 (201)	30.9 (38)	0.616
Enterococcus spp (%. *n*)	16.4 (99)	3.3 (4)	**<0**.**001**^a^
Other germs (%. *n*)	12.6 (76)	25.2 (31)	**<0**.**001**
Unidentified germ (%. *n*)	14.4 (87)	23.6 (29)	**0**.**011**
Echocardiography			
Type of lesion			
Vegetation (%. *n*)	89.1 (539)	78.9 (97)	**0**.**02**
Length of vegetation (mm)	12.1 ± 8.6	11.8 ± 7.7	0.771
Length of vegetation >10 mm (%. *n*)	42.3 (256)	39 (48)	0.5
Perivalvular complication (%. *n*)	16.5 (100)	34.1 (42)	**<0**.**001**
Perforation (%. *n*)	35 (169/483)	33.3 (37/111)	0.741
LVEF < 50% (%. *n*)	26.1 (158)	32.5 (40)	0.146
Treatment and outcome			
Theoretical indication for surgery (%, *n*)	73.9 (447)	82.1 (101)	0.056
Early surgery (%. *n*)	42.8 (259)	52 (64)	0.061
Emergency surgery (%. *n*)	6.1 (37)	4.1 (5)	0.524^a^
Urgent surgery (%. *n*)	13.2 (80)	14.6 (18)	0.676
Elective surgery (%. *n*)	23.5 (142)	33.3 (41)	**0**.**022**
In-hospital mortality (%. *n*)	14.5 (88)	9.8 (12)	0.16
Recurrence of endocarditis (%. *n*)	8.1 (49)	10.6 (13)	0.371

Continuous variables are expressed as mean ± 1 standard deviation. Categorical variables are expressed as percentage and number.

Values in bold indicates *p* < 0.05.

BAV-IE, bicuspid aortic valve infective endocarditis; NYHA, New York Heart Association; LVEF, left ventricular ejection fraction; TAV-IE, tricuspid aortic valve infective endocarditis.

^a^ Fisher exact test.

**Table 2:** Multivariate logistic regression analysis of parameters associated with bicuspid aortic valve IE in the overall study population.

Variables	OR (CI 95%)	*p*
Age	0.95 (0.93–0.97)	**<0**.**001**
Male gender	2.38 (1.26–4.48)	**0**.**008**
Hypertension	0.85 (0.46–1.56)	0.594
Diabetes	0.79 (0.34–1.8)	0.569
Previous myocardial infarction	0.91 (0.29–2.87)	0.868
Charlson comorbidity index (excluding age)	0.94 (0.78–1.13)	0.509
Major neurological event	1.85 (1.06–3.23)	**0**.**029**
Staphylococcus spp	0.63 (0.33–1.2)	0.159
Enterococcus spp	0.23 (0.07–0.7)	**0**.**01**
Other germ	1.71 (0.93–3.14)	0.085
Unidentified germ	1.95 (1.07–3.57)	**0**.**03**
Perivalvular complication	2.04 (1.24–3.36)	**0**.**005**

Values in bold indicates *p* ≤ 0.05.

There was a trend towards more theoretical indication for surgery (*p* = 0.056) and more early surgery in BAV-IE patients (*p* = 0.06) in whom, the absence of neurological events [OR(95%CI) = 0.27(0.10–0.92; *p* = 0.030)] and the presence of perivalvular complications [OR(95%CI) = 19.1(5.4–37.53; *p* < 0.001)] were independently associated with early surgery performance.

**Figure 1:**
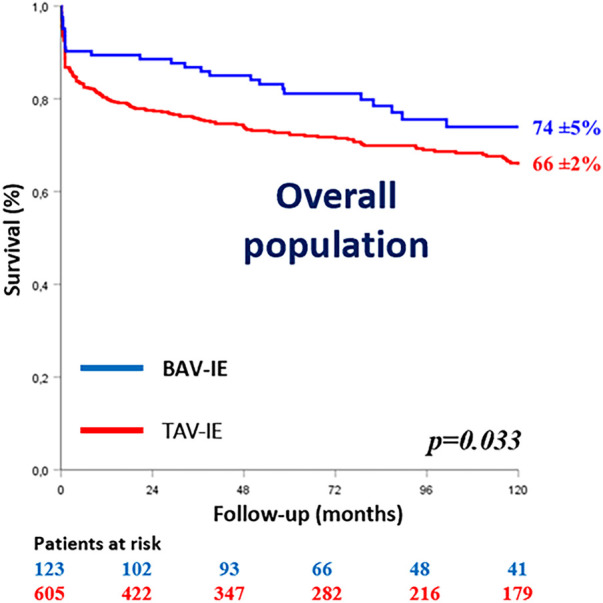
Kaplan–Meier survival curves of the study population according to the aortic valve anatomy. BAV-IE, bicuspid aortic valve infective endocarditis; TAV-IE, tricuspid aortic valve infective endocarditis.

In hospital mortality was comparable between the groups (9.8% for BAV-IE vs. 14.5% for TAV-IE; *p* = 0.16) but 10-year survival was better for patients with BAV-IE (74 ± 5% vs. 66 ± 2%; Log rank *p* = 0.033) (Figure 1). Only Staphylococcal infection was independently associated with in-hospital mortality in BAV-IE [OR(95%CI) = 4.33(1.70–17.50; *p* = 0.040)].

### Matched cohort

Differences in baseline characteristics according to aortic valve anatomy in the matched cohort are reported in Supplementary Table S1. After matching for age, sex and comorbidity (*n* = 109 in each group), BAV-IE had more embolic complications, fewer vegetations and more perivalvular complications (all *p* < 0.04). By multivariable logistic regression analysis, negative blood cultures [OR(95%CI) = 2.64(1.33–5.24); *p* = 0.005] and perivalvular complications [OR(95%CI) = 2.02(1.03–3.92); *p* = 0.041] were independently associated with BAV-IE (Supplementary Table S2).

**Figure 2:**
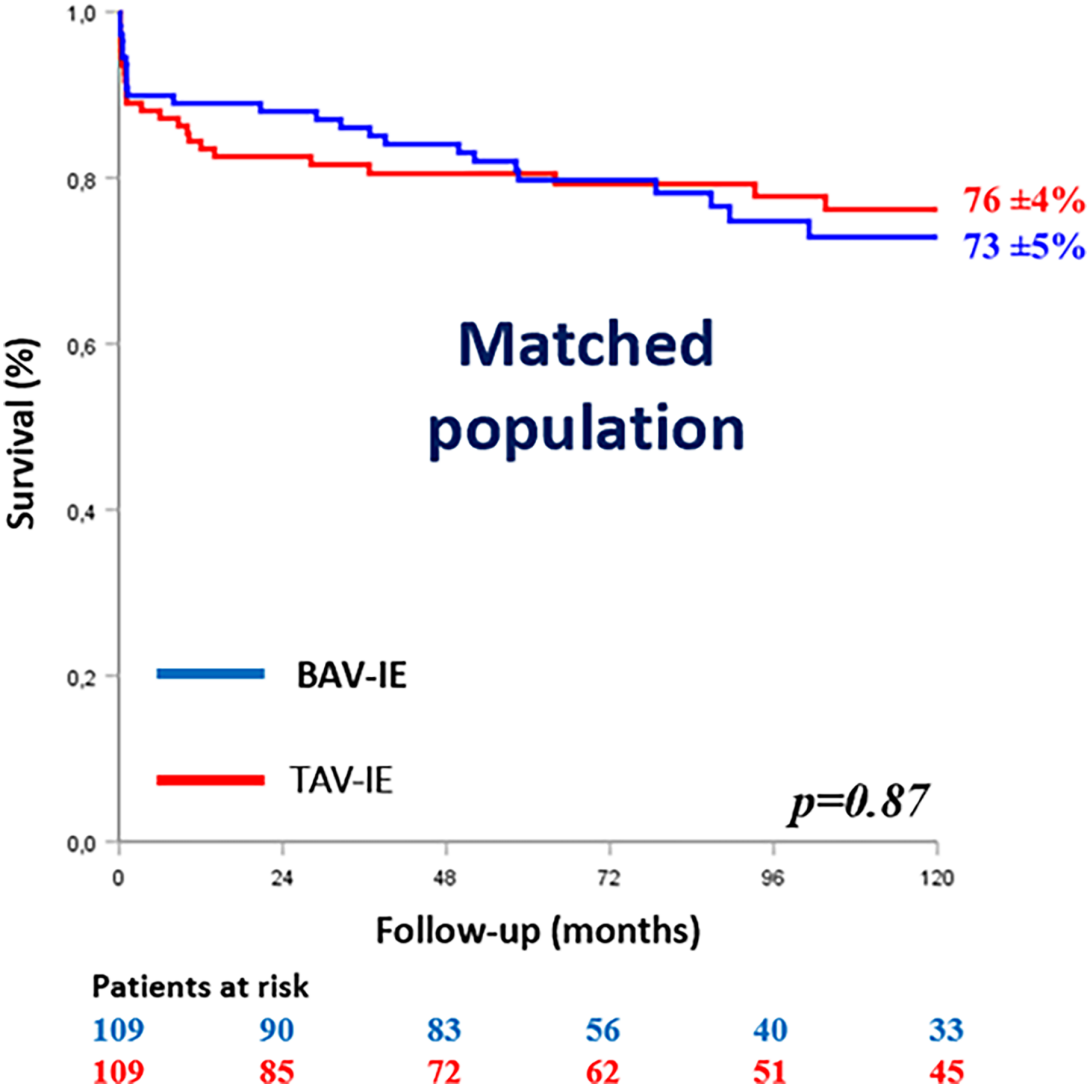
Kaplan–Meier survival curves of the matched population according to the aortic valve anatomy. BAV-IE, bicuspid aortic valve infective endocarditis; TAV-IE, tricuspid aortic valve infective endocarditis.

Theoretical indications for surgery (*p* = 0.72), early surgery (*p* = 0.41), in hospital mortality (*p* = 0.82) and estimated 10-year survival rates (Peto Logrank *p* = 0.87) were comparable between groups (Figure 2).

## Discussion

To our knowledge, this is the largest study comparing patients with isolated native aortic valve IE according to aortic valve anatomy. BAV was found in 16.9%, which agrees with previous reports suggesting a prevalence between 15% and 20% in aortic valve IE (2, 4). The population-based incidence of BAV-IE (definite and possible) is approximately 14 cases per 10,000 patient-years, 11 times higher than the general population, and appears greater than the incidence of aortic dissection for BAV patients (7). In addition, the rate of native valve IE in most adult BAV cohorts has been reported at approximately 2% across the board (4, 8). Therefore, IE is an important life-threatening complication in BAV patients. As expected, patients with BAV-IE were younger with few comorbidities.

Regarding bacteriological differences, there were no differences between staphylococcal and streptococcal infections, but enterococcal infections were more frequent in patients with TAV-IE, probably due to the fact that patients with TAV were older, with more comorbidities and more urinary and colonic infections. Patients with BAV-IE had more negative blood cultures than those with TAV-IE (23.6% vs. 14.4%). This 24% rate of negative blood cultures was already observed in our previous study (3) and is probably due to the prehospital empirical antibacterial treatment frequently administered in this young population unsuspected of bearing IE.

It has been established that the risk of embolism correlates with the size of the vegetations (>10 mm) and their mobility (9). In our study, there was no significant difference in vegetation length or embolic events between BAV-IE and TAV-IE. However, there were more major neurological events (22% vs. 14% respectively, *p* = 0.027) in bicuspid patients and the occurrence of a major neurological event was independently associated with BAV.As previously reported (2, 3), patients with BAV-IE had more perivalvular complications than those with TAV-IE. This susceptibility to perivalvular lesions remains poorly understood and we speculate that it could be related to the BAV-dependent abnormal blood flow impacting the aortic root. A more worrisome hypothesis, given that perivalvular complications develop late in the evolution of IE, is that diagnosis and referral may be late in this young population due to unsuspected IE. In addition, because abscesses usually develop late in the evolution of IE, it is possible that empirical antibiotic therapy administered before IE diagnosis contributed to delaying the diagnosis and the higher rate of culture negativity in BAV-IE.

The rate of early surgery in patients with BAV-IE was 52%, compared with 42.8% in patients with TAV-IE. These results were at the limit of statistical significance (*p* = 0.061). Lamas et al. (2) reported a higher rate of surgery for BAV-IE (90%) but their population was more severe, with more perivalvular complications. Indeed, in our study, the parameter most strongly associated with surgery was the presence of perivalvular complications.

In-hospital mortality was approximately 10% and was comparable to that of patients with TAV-IE, suggesting that despite their young age and low comorbidities, these patients do not carry a benign outcome. The better 10-year survival in BAV-IE is likely explained by this difference in age and comorbidity because this is no longer the case after matching for these variables.

The main limitations of the study are that it is retrospective and conducted over a long period of time, during which diagnostic methods, microbial ecology, antibiotic treatments and surgical management have evolved considerably. We cannot rule out the possibility that our population represent a selected cohort from referral centres with more severe disease.

BAV is a common finding in patients with isolated aortic valve IE and is associated with perivalvular complications in approximately one third of cases and a major neurological event in nearly one quarter of cases. Patients with BAV experienced a better survival at 10 years than patients with TAV-IE. However, the differences in survival are likely related to differences in age and comorbidities between these two populations and not to a more benign condition.

## Data Availability

The data analyzed in this study is subject to the following licenses/restrictions: The raw data supporting the conclusions of this article will be made available by the authors, without undue reservation. Requests to access these datasets should be directed to tribouilloy.christophe@chu-amiens.fr.
