# Supplementary material for: Characteristics and prognosis of isolated aortic valve infective endocarditis in patients with bicuspid aortic valves: a propensity matched study
Source: Front Cardiovasc Med. 2023 Dec 14;10:1304957. doi: 10.3389/fcvm.2023.1304957 (PMC10753019; doi:10.3389/fcvm.2023.1304957)
Supplement: Supplementary file 1 [file Datasheet1.docx]

**Supplemental Table 1: Baseline characteristics according to the aortic valve anatomy in the propensity matched cohort**

|  | **Tricuspid aortic valve IE**  **(109)** | **Bicuspid aortic valve IE**  **(109)** | **p** | **SMD** |
| --- | --- | --- | --- | --- |
| Age (years) | 51±14 | 51±14 | 0.98 | 0.01 |
| Male sex (%.n) | 86.2 (84) | 88.1 (96) | 0.84 | 0.05 |
| Charlson index (without age) | 1.6±1.4 | 1.6±1.3 | 0.91 | 0.02 |
| Congestive heart failure (%.n) | 36.7 (40) | 34.9 (38) | 0.89 | 0.04 |
| Embolic complications (%.n) | 42.2 (46) | 56.0 (61) | 0.040 | 0.28 |
| Neurological events (%.n) | 22.9 (25) | 20.2 (22) | 0.74 | 0.06 |
| Negative blood cultures (%.n) | 25.7 (28) | 15.6 (17) | 0.094 | 0.25 |
| Vegetations (%.n) | 78.0 (85) | 92.6 (100) | 0.004 | 0.33 |
| Perivalvular complications (%.n) | 20.2 (22) | 33.0 (36) | 0.032 | 0.30 |
| Early surgery (%.n) | 58.7 (64) | 53.2 (58) | 0.41 | 0.09 |
| In hospital mortality (%.n) | 10.1 (11) | 11.0 (12) | 0.82 | 0.03 |

IE: infective endocarditis, SMD: standardized mean difference

Supplementary Table 2 : Multivariate logistic regression analysis of parameters associated with bicuspid aortic valves in the overall study population.

| **Variables** | **OR (CI 95%)** | **p** |
| --- | --- | --- |
| Age  Male gender  Hypertension  Diabetes  Previous myocardial infarction  Charlson comorbidity index (excluding age)  Major neurological event  Staphylococcus spp  Enterococcus spp  Unidentified germ  Perivalvular complication | 1.00 (0.98-1.02)  0.93 (0.40-2.18)  0.67 (0.30-1.46)  0.72 (0.24-2.18)  0.43 (0.09-1.99)  1.08 (0.83-1.42)  1.51 (0.74-3.07)  0.76 (0.34-1.66)  0.34 (0.10-1.21)  2.64 (1.33-5.24)  2.02 (1.03-3.92) | 0.673  0.870  0.312  0.564  0.280  0.546  0.256  0.491  0.096  **0.005**  **0.041** |
|  | | |
